# The immune receptor CD300e negatively regulates T cell activation by impairing the STAT1-dependent antigen presentation

**DOI:** 10.1038/s41598-020-73552-9

**Published:** 2020-10-05

**Authors:** Sara Coletta, Valentina Salvi, Chiara Della Bella, Ambra Bertocco, Silvia Lonardi, Elisabetta Trevellin, Matteo Fassan, Mario M. D’Elios, William Vermi, Roberto Vettor, Stefano Cagnin, Silvano Sozzani, Gaia Codolo, Marina de Bernard

**Affiliations:** 1 Department of Biology, University of Padova, Via Ugo Bassi 58/B, 35131 Padova, Italy; 2 Department of Molecular and Translational Medicine, University of Brescia, Brescia, Italy; 3 Department of Experimental and Clinical Medicine, University of Florence, Florence, Italy; 4 Department of Molecular and Translational Medicine, Section of Pathology, University of Brescia, Brescia, Italy; 5 Department of Medicine, Clinica Medica 3a, Azienda Ospedaliera Di Padova, University of Padova, Padova, Italy; 6 CRIBI Biotechnology Center, University of Padova, Padova, Italy; 7 Laboratory Affiliated To Istituto Pasteur Italia-Fondazione Cenci Bolognetti, Department of Molecular Medicine, Sapienza University of Rome, Rome, Italy

**Keywords:** Cell biology, Immunology

## Abstract

CD300e is a surface receptor, expressed by myeloid cells, involved in the tuning of immune responses. CD300e engagement was reported to provide the cells with survival signals, to trigger the expression of activation markers and the release of pro-inflammatory cytokines. Hence, CD300e is considered an immune activating receptor. In this study, we demonstrate that the ligation of CD300e in monocytes hampers the expression of the human leukocyte antigen (HLA) class II, affecting its synthesis. This effect, which is associated with the transcription impairment of the signal transducer and activator of transcription 1 (STAT1), overcomes the capacity of interferon gamma (IFN-γ) to promote the expression of the antigen-presenting molecules. Importantly, the decreased expression of HLA-II on the surface of CD300e-activated monocytes negatively impacts their capacity to activate T cells in an antigen-specific manner. Notably, unlike in vitro- differentiated macrophages which do not express CD300e, the immune receptor is expressed by tissue macrophages. Taken together, our findings argue against the possibility that this molecule should be considered an activating immune receptor sensu stricto. Moreover, our results support the notion that CD300e might be a new player in the regulation of the expansion of T cell-mediated responses.

## Introduction

CD300e, originally termed immune receptor expressed by myeloid cells (IREM)-2, is a glycosylated surface receptor with a single extracellular Ig-like domain, belonging to a family of immune receptors that includes 8 members, either activating or inhibitory, that are expressed on myeloid cells, on lymphoid cells or on both compartments^1^. Since the ligand of CD300e is still unknown, its function in human cells has been studied by using an agonistic anti-CD300e monoclonal antibody^2^. The evidence that the engagement of CD300e in human monocytes and myeloid dendritic cells provided the cells with survival signals and triggered the expression of the activation markers and the release of pro-inflammatory cytokines, led to the conclusion that CD300e is an immune-activating receptor^2^. The presence of a lysine residue in the transmembrane domain suggested that CD300e might associate with an ITAM-containing adaptor molecule. In accordance, CD300e was shown to associate with DNAX-activating protein 12 (DAP12) in COS-7 transfected cells^3^. However, such an interaction was not confirmed in primary human monocytes and the signalling pathway triggered by CD300e remains elusive.

Another study, focused on the CD300e ortholog in mice, came to similar conclusions^4^. In mouse CD300e is highly expressed only in nonclassical (CD14^low^CD16^+^) and intermediate (CD14^+^CD16^+^) monocytes, while the human counterpart is expressed in all subsets of human monocytes, including the classical one (CD14^+^CD16^−^). However, intermediate and nonclassical monocytes exhibit slightly higher expression levels^5^. CD300e was found also highly expressed in a recently identified murine splenic myeloid antigen-presenting cells, labelled as L-DC^6^.

In vitro differentiation of monocytes to macrophages is accompanied by the down-regulation of CD300e expression^3^. However, we recently reported that the expression of the immune receptor in monocyte-derived macrophages can be rescued by the down-modulation of miR-4270^7^. In addition, Pagliari and colleagues revealed that the activation of rescued-macrophages by the CD300e agonistic antibody, led to a decreased exposure of HLA-DR on the plasma membrane^7^.

These data raised the intriguing possibility that CD300e might elicit both an activating and an inhibitory pathway, and prompted us to investigate in human monocytes, a cell subset that strongly expresses both CD300e and HLA-II molecules, the mechanisms used by CD300e to hinder the expression of HLA-II molecules.

## Methods

### Monocyte isolation and cell treatment

Monocytes were purified from buffy coats of healthy donors by density gradient protocol, as described previously^8^. Alternatively, monocytes were purified by negative selection using Classical Monocyte Isolation Kit (Miltenyi Biotec, Bergisch Gladbach, North Rhine-Westphalia, Germany), following the manufacturer’s instructions. Cells were maintained in RPMI 1640 10% FBS, 50 μg/ml gentamicin and 4 mM HEPES.

To evaluate the impact of the activation through CD300e, cells were plated at a density of 2 × 10^6^ per well in 24-well plates previously coated with 200 μl of PBS containing 10 µg/ml of monoclonal antibody anti-CD300e (clone UP-H2, kindly provided by M. López-Botet, Barcelona, Spain) or isotype-matched control (IgG_1_, MOPC-21, Abcam, Cambridge, UK), for 24 h (unless differently specified).

Cross-linking of CD64 was performed as described elsewhere^9^. Briefly, 24-well plates were coated with 50 µg/ml of goat anti-mouse Fc-specific F(ab′)_2_ fragment (Sigma-Aldrich, St. Louise, MO, USA) in 200 μl of PBS overnight at 4 °C. Wells were then coated with 10 µg/ml of anti-CD64 (Clone 10.1, Ebiosciences, San Diego, CA, USA) or control IgG_1_ monoclonal antibody in 200 μl of PBS + 2.5% BSA for 2 h at 37 °C. 2 × 10^6^ monocytes were added to each well and incubated for 24 h at 37 °C.

Referring to the experiments with conditioned media, monocytes were treated as above. After 24 h, media were collected, centrifuged and transferred on freshly isolated monocytes seeded on 24-well plate. Antibody-depleted media were obtained by incubating 1.5 ml of conditioned medium with 10 µl of protein G agarose (ThermoFisher, Waltham, MA, USA) at RT for 2 h, in rotation. Cells were processed for subsequent analyses 24 h after receiving media.

For the experiments including IFN-γ stimulation, monocytes were activated with anti-CD300e, as above. After 6 or 16 h, medium was changed with a fresh one containing 10 ng/ml IFN-γ. After 6 or 8 h, cells were processed for qRT-PCR and Flow Cytometry. Six h of activation with anti-CD300e antibody plus 6 h with IFN-γ was the timing adopted for qRT-PCR, while 16 h plus 8 h was the timing adopted for Flow Cytometry. For the evaluation of pSTAT1 by western blot, cells were harvested at 5, 10 and 15 min after receiving IFN-γ-containing medium.

When indicated, monocytes were simultaneously stimulated with 10 ng/ml IFN-γ and 10 µg/ml anti-CD300e and harvested after 5, 10 and 15 min for the evaluation of pSTAT1 by western blot.

### Patients

Cells and tissues were collected from patients in accordance with the principles of the Helsinki Declaration and written informed consent was obtained from each patient, according to the study protocol approved by the Ethical Committee of the Universities of Padova, Brescia and Florence.

### Isolation of stromal vascular fraction (SVF) from adipose tissue

SVF was isolated from subcutaneous adipose tissue of 3 obese patients, who underwent plastic surgery (abdominoplasty). Briefly, freshly collected subcutaneous adipose tissue was separated from major vessels and fibers, minced and digested with 1 mg/ml collagenase type II (Sigma-Aldrich) in DMEM-F12 (ThermoFisher) at 37 °C for 1 h. Cell suspension containing SVF was centrifuged (350×*g*, 8 min), pellet was resuspended in erythrocyte-lysing buffer for 5 min, washed in DMEM-F12 3% FBS, filtered with a 100 μm cell strainer, centrifuged (350×*g*, 8 min) and resuspended in DMEM-F12 10% FBS for further analysis.

### Flow cytometry

#### Monocytes

Cells were harvested from culture plates using 5 mM Na-EDTA in PBS pH 7.5 and incubated for 15 min at RT with 10% human serum in FACS buffer (PBS, 1% BSA) to saturate Fc receptors. Depending on the experiment, 1 × 10^6^ cells were stained with combinations of the following antibodies: PerCP-Cyanine5.5-conjugated anti-CD14 (clone 61D3, Ebiosciences), FITC-conjugated anti-CD16 (clone 3G8, BD Biosciences, San Jose, CA,USA), APC-conjugated anti-HLA-DR (clone L243, Ebiosciences)^10^ or with PECyanine7-conjugated anti-CD86 (clone B7-2, Ebiosciences), PE-conjugated anti-CD68 (clone Y1/82A, Ebiosciences), BB515-conjugated anti-CD206 (clone 19.2, Ebiosciences), PerCP-Cyanine5.5-conjugated anti-CD163 (clone GHI/61, Ebiosciences), FITC-conjugated anti-HLA-DQ (clone TU169 , BD Biosciences), unconjugated anti-HLA-DP (clone B7/21, Abcam) followed by a goat anti-mouse Alexa Fluor 488 antibody (ThermoFisher). The fixable cell viability dye eFluor780 (Ebiosciences) was used to exclude dead cells from the analysis. For the intracellular staining of CD68, monocytes were fixed with 3.7% formaldehyde and permeabilized with PermWash solution (1% FBS, 0.2% saponin in PBS). Cells were resuspended in FACS buffer and analysed by FACSCanto II (Becton Dickinson, Franklin Lakes, NJ, USA). Values were expressed as *n*-fold change of median fluorescence intensity (MFI) vs T_0_ or untreated cells. Data were analysed using FlowJo software, version 10.3 (Tree Star Inc., Ashland, OR, USA).

#### Stromal vascular fraction (SVF)

Cells were resuspended in FACS buffer and incubated for 15 min at RT with 10% human serum to saturate Fc receptors. 1 × 10^6^ cells were stained with the monoclonal antibody anti-CD300e, followed by a goat anti-mouse Alexa Fluor 488 antibody (ThermoFisher). The cell viability dye eFluor780 (Ebiosciences) was used to exclude dead cells from the analysis. Cells were fixed with 3.7% formaldehyde and permeabilized with PermWash solution (1% FBS, 0.2% saponin in PBS). Cells were stained with PE-conjugated anti-CD68 monoclonal antibody, resuspended in FACS buffer and analysed by FACSCanto II. Forward and side scatter light were used to identify cell populations. Values were expressed as the percentage of CD300e positive cells inside the gate of CD68 positive cells. A sample labelled only with the goat anti-mouse Alexa Fluor 488 antibody (ThermoFisher) was used to define the background. The percentage of cells expressing CD300e with MFI ≤ of background was considered negative. Based on the evidence that the intensity of CD300e expression identified two distinct CD68-positive cell populations, we arbitrarily defined them as CD300e^bright^ and CD300e^dim^. All data were analysed using FlowJo software.

### Flow cytometry-based internalization and recycling assay

For the internalization assay, monocytes were incubated with biotinylated anti-HLA-DR (clone L243, BioLegend, San Diego, CA, USA) in chilled FACS buffer. After 30 min, cells were washed and incubated in RPMI 10% FBS at 37 °C in rotation, in presence of medium alone, anti-CD300e antibodies or control IgG. The amount of tagged-HLA-DR remaining on the cell surface was determined by incubating the cells with Alexa 488-conjugated streptavidin (ThermoFisher) on ice. Cells were analysed by flow cytometry and the MFI at each time point was expressed as % of the value at time 0 (set as 100%). HLA-DR recycling assay was performed incubating monocytes with biotinylated anti-HLA-DR mAb (clone L243, BioLegend) at 37 °C for 30 min. Cells were washed in ice-cold FACS buffer and the surface-exposed antibodies were blocked using the Alexa 555-conjugated streptavidin (ThermoFisher). Cells were washed in FACS buffer and then cultured in pre-warmed complete medium at 37 °C in rotation, in presence of medium alone, control IgG or anti-CD300e for indicated time points. The recycled HLA-DR reappeared on the cell surface was detected by staining the cells with Alexa 488-conjugated streptavidin (ThermoFisher) on ice. Cells were analysed by flow cytometry and the MFI at each time point was expressed as % of the value at 120 min (set as 100%).

### Western blot

Western blot was performed as reported elsewhere^7^. Briefly, monocytes were lysed in RIPA buffer (50 mM Tris–HCl, pH 7.4, 150 mM NaCl, NP-40 1%, Na-deoxycholate 0.5%, SDS 0.1%, 2 mM EDTA, 50 mM NaF, 1 mM Na_3_VO_4_, 1 mM EGTA, 2% PMSF) with protease inhibitor cocktail (Millipore, Burlington, MA, USA), and proteins were quantified by BCA protein assay kit (ThermoFisher), according to the manufacturer’s instructions. Five micrograms of proteins from each sample were resuspended in NuPAGE LDS sample buffer (Novex, Life Technologies, Waltham, MA, USA) supplemented with 50 mM DTT and denaturated for 5 min at 100 °C. Proteins were separated electrophoretically in NuPAGE Bis-Tris 4-12% polyacrylamide gel (Novex, Life Technologies), and subsequently transferred on PVDF membranes (Millipore). Membranes were blocked with 5% BSA (Sigma-Aldrich) in Tris-buffered saline (TBS, 50 mM Tris–HCl pH 7.6, 150 mM NaCl) containing 0.1% Tween20 (Sigma-Aldrich), and antigens were revealed using the following antibodies: mouse anti-human HLA-DR α-chain monoclonal antibody (1:5000, clone TAL-1B5 Abcam), rabbit anti-STAT1 polyclonal antibody (1:1000, Cell Signaling Technology, Danvers, MA, USA), rabbit anti-phosphoSTAT1 (Tyr701) monoclonal antibody (1:1000, Cell Signaling Technology), rabbit anti-vinculin polyclonal antibody (1:3000, Origene, Rockville, MD , USA), mouse anti-β-actin (1:10,000, Sigma-Aldrich). Blots were washed three times with TBS plus 0.1% Tween20 and incubated for 1 h at RT with horseradish peroxidase-conjugated anti-rabbit (Millipore) or anti-mouse IgG secondary antibody (Novex, Life Technologies). Blots were developed with enhanced chemiluminescence substrate (EuroClone, Pero, MI, Italy), and the protein bands were detected using ImageQuant LAS 4000 (GE Healthcare Life Science, Chicago, IL, USA).

### ELISA

Culture supernatants of stimulated monocytes were collected and stored at − 80 °C for subsequent quantification of cytokine content. ELISA kits, specific for IL-1β, IL-6, IL-8 and TNF-α (eBiosciences), were used following the manufacturers’ instructions.

### T-cell proliferation assay

Monocytes isolated by CD14 positive selection (CD14 MicroBeads, Mylteni Biotec) from 10 patients with melanoma, were seeded as above on plates coated with anti-CD300e mAb or with isotype IgG. After 24 h of stimulation, cells were harvested and co-cultured in triplicate with CD4^+^ T lymphocytes, purified from the same patients, in presence of the melanoma associated antigen 3 (MAGE-A3), for 5 d. At 16 h before harvesting, 1 μCi/well of ^3^[H]-TdR was added and the radionuclide uptake was measured, as reported^11^.

### RNA extraction and qRT-PCR

Total RNA was extracted with TRIzol reagent (ThermoFisher) according to the manufacturer’s protocol. One μg of total RNA was retrotranscribed using the High-Capacity cDNA Reverse Transcription Kit (Applied Biosystems, Waltham, MA, USA), following the manufacturer’s instructions. Five ng of cDNA obtained was used in qRT-PCR reaction performed with PowerUp SYBR Green (Applied Biosystems). For each sample, data were normalized to the endogenous reference gene β-actin. Sequence of primers used are listed as follows. β-actin forward 5′-TGAGATGCGTTGTTACAGGA-3′, reverse 5′-ACGAAAGCAATGCTATCA-3′; HLA-DR α chain forward 5′-GCCCTGTGGAACTGAGAGAG-3′, reverse 5′-CTGGTGGGGTGAACTTGTCT-3′; HLA-DR β chain forward 5′-AGGCAGCATTGAAGTCAGGT-3′, reverse 5′-ATTCTGAATCAGGCCTGTGG-3′; CIITA forward 5′-GGTCCAGGGTTTGAGTTCAT-3′, reverse 5′-TGATTTGGGGTGGCTTGTTA-3′; STAT1 forward 5′-GGCAAAGAGTGATCAGAAACAA-3′, reverse 5′-GTTCAGTGACATTCAGCAACTC-3′; HLA-DP α chain forward 5′-CTCTGTTGCCGATGTGGTTA-3′, reverse 5′-TCCTCAGACCTTTCCGTTCA-3′; HLA-DP β chain forward 5′-TGCCCCCAAATCAAGTTTAG-3′, reverse 5′-GCAGTCTGCTCACCATTGAA-3′; HLA-DQ α chain forward 5′- TCCCCTCAGAGCTCACAAAT-3′, reverse 5′-ACCCCAGGCATGTCTTTGTA-3′; HLA-DQ β chain forward 5′-CAGCCTCTCTTCTGGTTTGG-3′, reverse 5′-TATCTGCCCCCAAACAATTC-3′. Data analysis was carried on according to ΔΔCt method.

### Pulse biosynthetic labelling

Monocytes, stimulated for 24 h with anti-CD300e mAb or isotype IgG, were incubated 1 h in Met-free medium (ThermoFisher) to deplete methionine reserves. Cells were labelled with the methionine analogue L-Azidohomoalanine (AHA 50 µM, Click-IT Invitrogen, Waltham, MA, USA) for 2 h and lysed in 50 mM Tris–HCl, pH 8.0, 1% SDS. All newly synthetized proteins were tagged with biotin alkyne (Click-IT Invitrogen) according to the manufacturer’s protocol. Proteins were pulled-down with NeutrAvidin agarose resin (Pierce, Waltham, MA, USA), processed for western blot, and developed for anti-human HLA-DR α-chain monoclonal antibody (1:5000, clone TAL-1B5 Abcam).

### Immunohistochemistry

Immunohistochemistry was performed as reported elsewhere^7^. Briefly, formalin-fixed paraffin embedded tissue sections were stained with a rabbit anti-CD300e polyclonal antibody (Sigma-Aldrich). On appropriate antigen retrieval (water bath at 98 °C for 40 min in ethylenediaminetetraacetic buffer pH 8.0), reactivity was revealed using NovoLink Polymer horseradish peroxidase-linked (Leica Biosystems, Wetzlar, Germany) followed by Diaminobenzidine (DAB). Analysis of CD300e positive cells was performed by double immunohistochemistry. After completing the first immune reaction, the second was realized using a monoclonal primary antibody to CD163 (clone 10D6, ThermoFisher), visualized using Mach 4-AP (Biocare Medical, Pacheco, CA, USA), followed by Ferangi Blue (Biocare Medical) as chromogen. Quantification of positive cells was performed on 10 high power fields (HPF) on sections double stained for CD300e and CD163. Immunostained sections were photographed using the DP-70 Olympus digital camera mounted on the Olympus BX60 microscope, and the digital pictures (each corresponding to 0.13 mm^2^) were used for cell count. Values were expressed as mean percentage of CD300e positive cells inside the CD163 positive cells. Based on the intensity of CD300e expression, CD163 positive cells were classified as negative, CD300e^bright^ and CD300e^dim^.

## Results

### CD300e activation reduces HLA-II total cell content in monocytes

**Figure 1:**
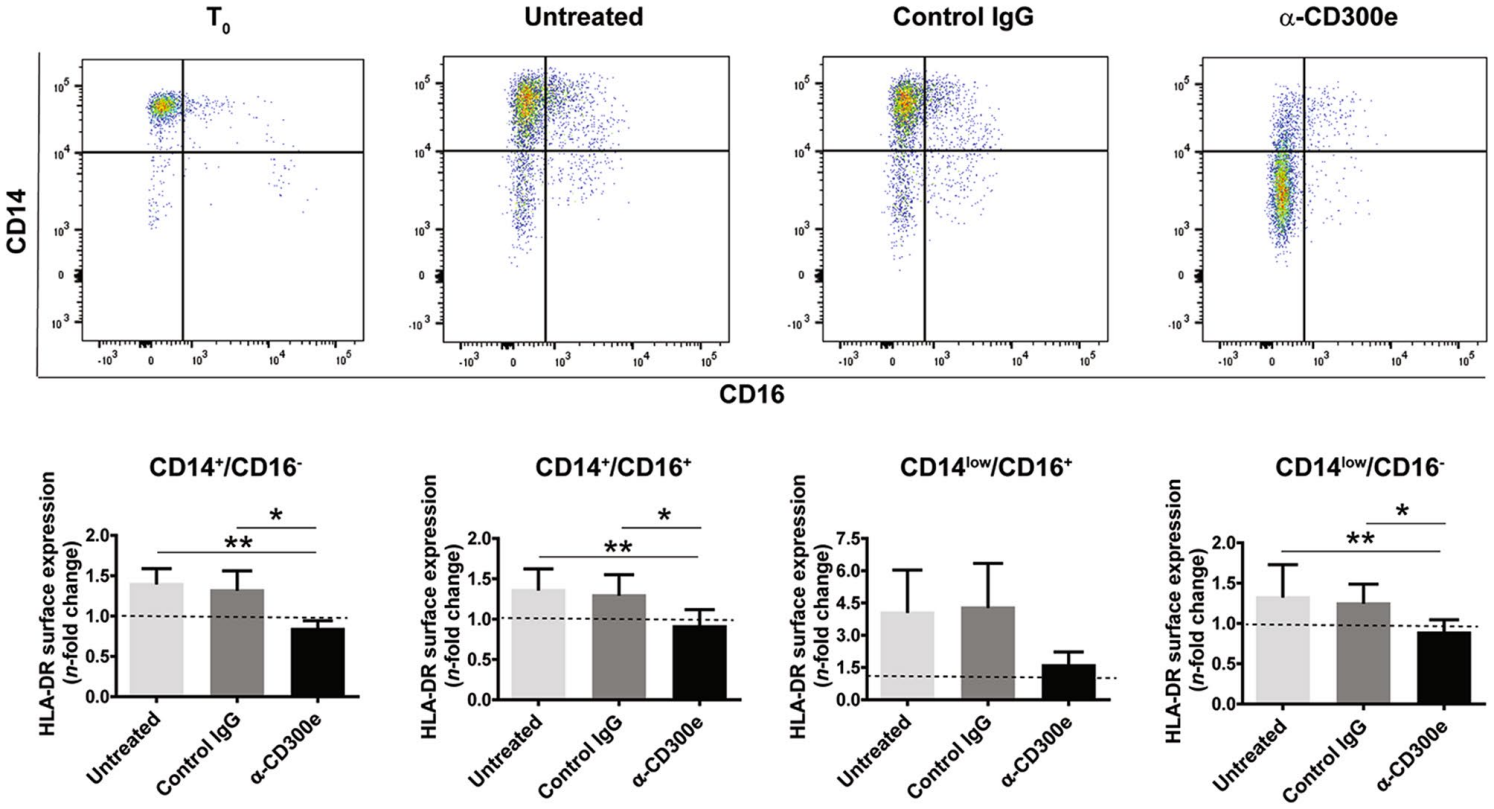
Activation through CD300e reduces the expression of HLA-DR in all monocyte subsets. Freshly isolated monocytes were plated on 24-well plates uncoated, coated with isotypic IgG (control IgG) or with anti-CD300e monoclonal IgG (UP-H2). At time 0 and after 24 h cells were labelled with anti-CD14, anti-CD16 and anti-HLA-DR antibodies. The expression level of CD14 and CD16 was used to identify 4 cell subsets, namely CD14^+^CD16^−^, CD14^+^CD16^+^, CD14^low^CD16^+^ and CD14^low^CD16^−^. Upper panels: representative cytograms. Lower panels: median fluorescence intensity (MFI) of HLA-DR ± SEM of 4 independent experiments was calculated for each subset. Expression levels of HLA-DR are expressed as *n*-fold change relative to time 0 (T_0_) set as 1 (dotted line). Significance was determined by Student’s *t*-test. *p ≤ 0.05; **p ≤ 0.01.

Based on the evidence that the activation through CD300e affected the capacity of macrophages to expose HLA-DR antigens on the plasma membrane^7^, we wondered whether this pattern was recapitulated in monocytes which, unlike in vitro-differentiated macrophages, highly express the immune receptor^2^. Monocytes purified from buffy coat by density gradient were stimulated with the monoclonal antibody agonistic for CD300e (clone UP-H2)^3^. After 24 h we determined the expression of HLA-DR in all monocytes subsets, CD14^+^CD16^−^ (classical), CD14^+^CD16^+^ (intermediate), and CD14^low^CD16^+^ (nonclassical). In line with our previous observations, with respect to monocytes exposed to the isotype-matched control, the engagement of CD300e maintained the amount of HLA-DR low in all subsets, even if for nonclassical monocytes the effect did not reach the significance (Fig. 1). The low expression of HLA-DR was reproduced in classical monocytes purified by negative selection (data not shown). The latter result ruled out the possibility that the reduction of HLA-DR was due to remaining lymphocytes.

Twenty-four h after purification, among the cells seeded on uncoated or antibody-coated plate and identified as CD16^−^, a proportion become CD14^low^, as reported elsewhere^12^. We verified that in this subset the expression of HLA-DR was also kept low by CD300e engagement. It is noteworthy that the proportion of CD14^low^CD16^−^ cells was much higher in monocytes activated through CD300e than in control cells (Fig. 1). This effect, which certainly deserves further investigation, suggested that CD300e might be involved in monocyte differentiation towards a macrophage- or dendritic cell-like phenotype^12^.

According to the finding that the activation of monocytes through CD300e maintained at basal level the expression of HLA-DR on the plasma membrane of all monocyte subsets, we decided to perform the subsequent experiments without distinguishing among the subsets. Similar to HLA-DR, HLA-DP and -DQ antigens were also affected by the crosslinking of the immune receptor (Supplementary Fig. S1a,c).

In spite of the drop of HLA-II and in accordance with former studies^2,4^, activated monocytes up-regulated the co-stimulatory molecule CD86 and released pro-inflammatory cytokines (Supplementary Fig. S2). The increased expression of CD68 and CD206, further supported the hypothesis on the role of CD300e in promoting the differentiation of monocytes towards a macrophage-like phenotype (Supplementary Fig. S2). Oddly, the expression of the macrophage marker CD163 was unaffected following the activation through the immune receptor (data not shown).

### CD300e compromises the ability of monocytes to elicit an HLA-II-restricted T-cell response

**Figure 2:**
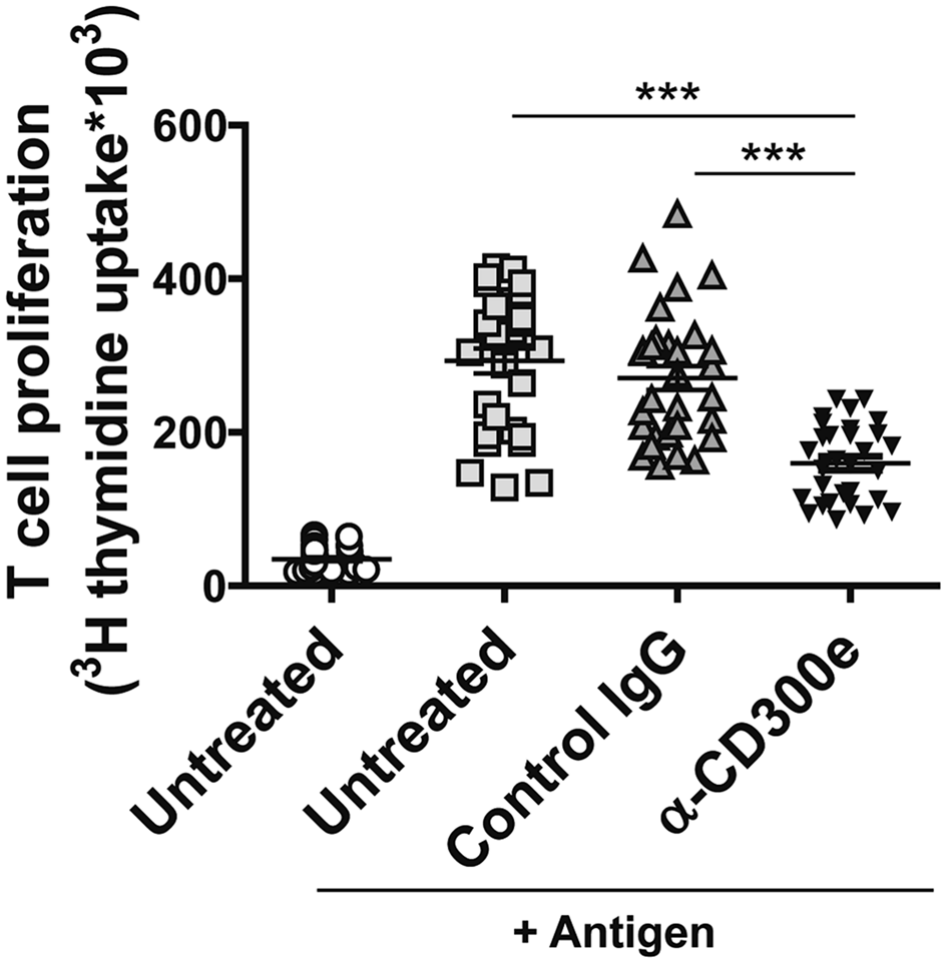
Activation through CD300e compromises the ability of monocytes to activate antigen-specific T cells. Monocytes isolated from 10 patients with melanoma were seeded in triplicate as above on uncoated plates or on plates coated with isotype IgG or anti-CD300e. After 24 h, cells were harvested and co-cultured with CD4^+^ T lymphocytes purified from the same patients in presence of specific melanoma antigen (MAGE-A3). Lymphocyte proliferation was measured by ^3^[H]-TdR incorporation. Data are shown as mean ± SEM of 10 independent experiments. Significance was determined by Student’s *t*-test. ***p ≤ 0.001.

We moved to investigate whether the down-regulation of HLA-II was sufficient to hamper the ability of monocytes to stimulate the antigen-specific proliferation of CD4^+^ T lymphocytes, despite the upregulation of the co-stimulatory molecule CD86^2^ (Supplementary Fig. S2). Monocytes isolated from melanoma patients were activated by the anti-CD300e antibody for 24 h and then used as antigen presenting cells towards autologous T lymphocytes, in presence of the melanoma antigen MAGE-A3^13^. As shown in Fig. 2, while monocytes, either untreated or exposed to the isotypic antibody, efficiently activated T cells, the ability of CD300e-ligated monocytes to induce T cell proliferation was significantly impaired.

### CD300e does not interfere with the intracellular trafficking of HLA-II

**Figure 3:**
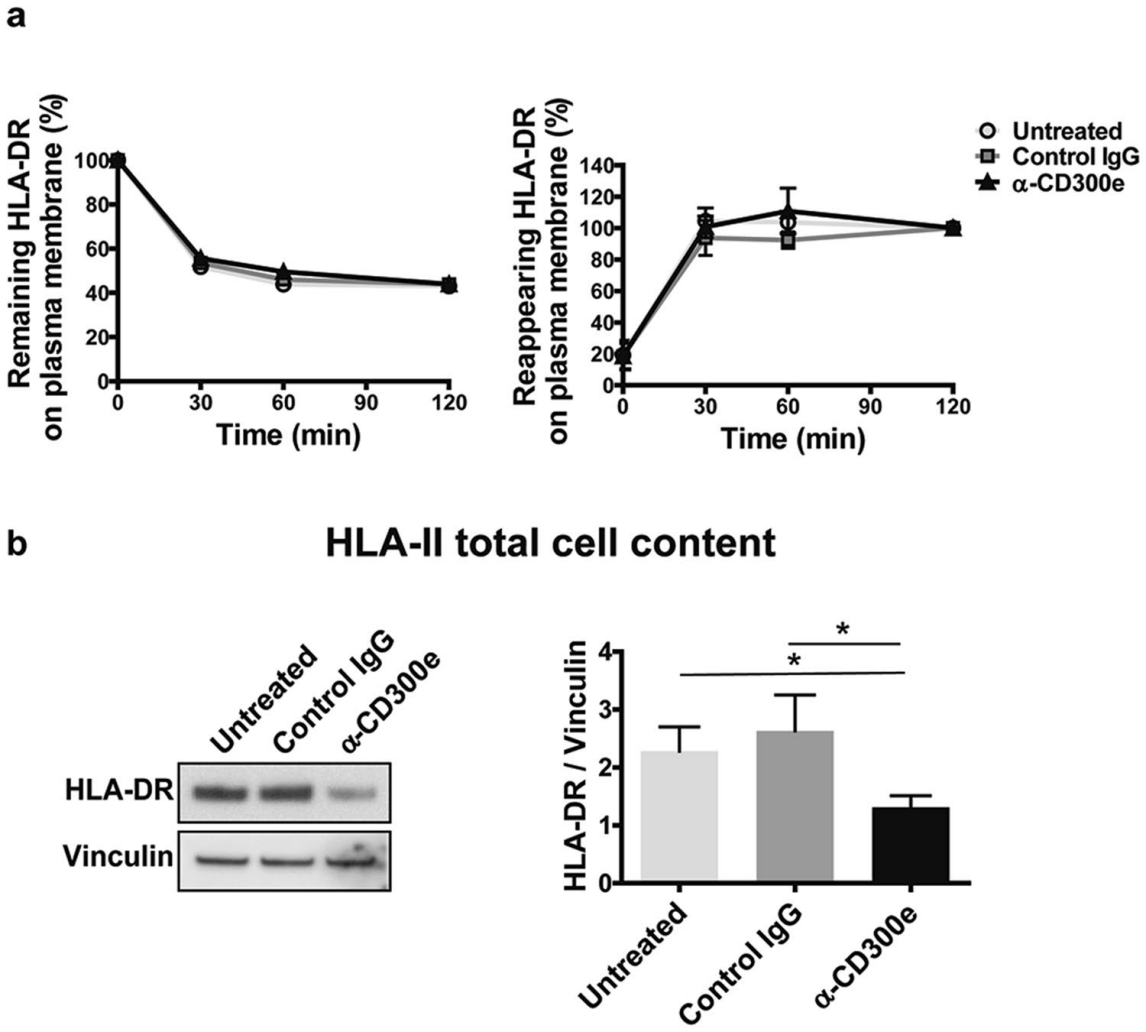
Activation through CD300e does not interfere with the intracellular trafficking of HLA-DR. (**a**) Kinetic of internalization and recycling of HLA-DR in monocytes. The amount of HLA-DR remaining or recycling back to the cell surface in untreated monocytes and in monocytes exposed to control IgG or anti-CD300e was determined by flow cytometry. For each sample, MFI values are expressed as percentage relative to T_0_ or to time 120 min (set as 100%). Data are shown as mean ± SEM of 5 independent experiments. (**b**) HLA-DR total cell content evaluated by western blot in monocytes seeded on uncoated, coated with isotypic IgG or with anti-CD300e for 24 h. Blot refers to a representative of 4 independent experiments. Quantification of HLA-DR was performed by densitometry and normalized to vinculin (mean ± SEM). Significance was determined by Student’s t-test. *p ≤ 0.05.

The expression of HLA-II on the surface of antigen presenting cells is not a static phenomenon, since the complexes are internalized and can recycle back from early endosomes to the cell surface, reaching an equilibrium^14,15^. Therefore, we hypothesized that the activation through the immune receptor could reduce the exposure of HLA-II by interfering with its intracellular trafficking. To explore this possibility, we examined the kinetics of HLA-DR internalization and recycling by flow cytometry-based assays^10^. In control monocytes, either untreated or exposed to isotype IgG, HLA-DR internalized rapidly and reached an equilibrium of 50% internalization after 60 min (Fig. 3a). The assessment of the HLA-DR recycling in control monocytes confirmed that the internalized molecules could traffic from intracellular compartments to the plasma membrane (Fig. 3a). Essentially identical results were obtained when examining the kinetics of internalization and recycling of HLA-DR in CD300e-activated monocyte. These findings ruled out the possibility that CD300e interferes with the trafficking of HLA-DR complexes.

We reasoned about the possibility that the ligation of CD300e could impact on HLA-DR expression. As revealed by western blot, the total amount (membrane + intracellular) of HLA-DR in stimulated monocytes, was actually reduced by ~ 50% (Fig. 3b).

We verified that the drop in HLA-DR expression did not depend on factors released in the medium (Supplementary Fig. S3). Moreover, the evidence that the crosslinking of Fc γ receptor I (CD64), which is also an activating receptor, did not influence HLA-DR expression, further disclosed the distinctive features of CD300e (Supplementary Fig. S3).

### CD300e affects the synthesis of HLA-II in monocytes

Based on the notion that class II major histocompatibility complex transactivator (CIITA) is the master regulator for the expression of HLA-II genes^16^, we assessed whether the abatement of HLA-II molecules in monocytes might be due to the down-regulation of CIITA.

**Figure 4:**
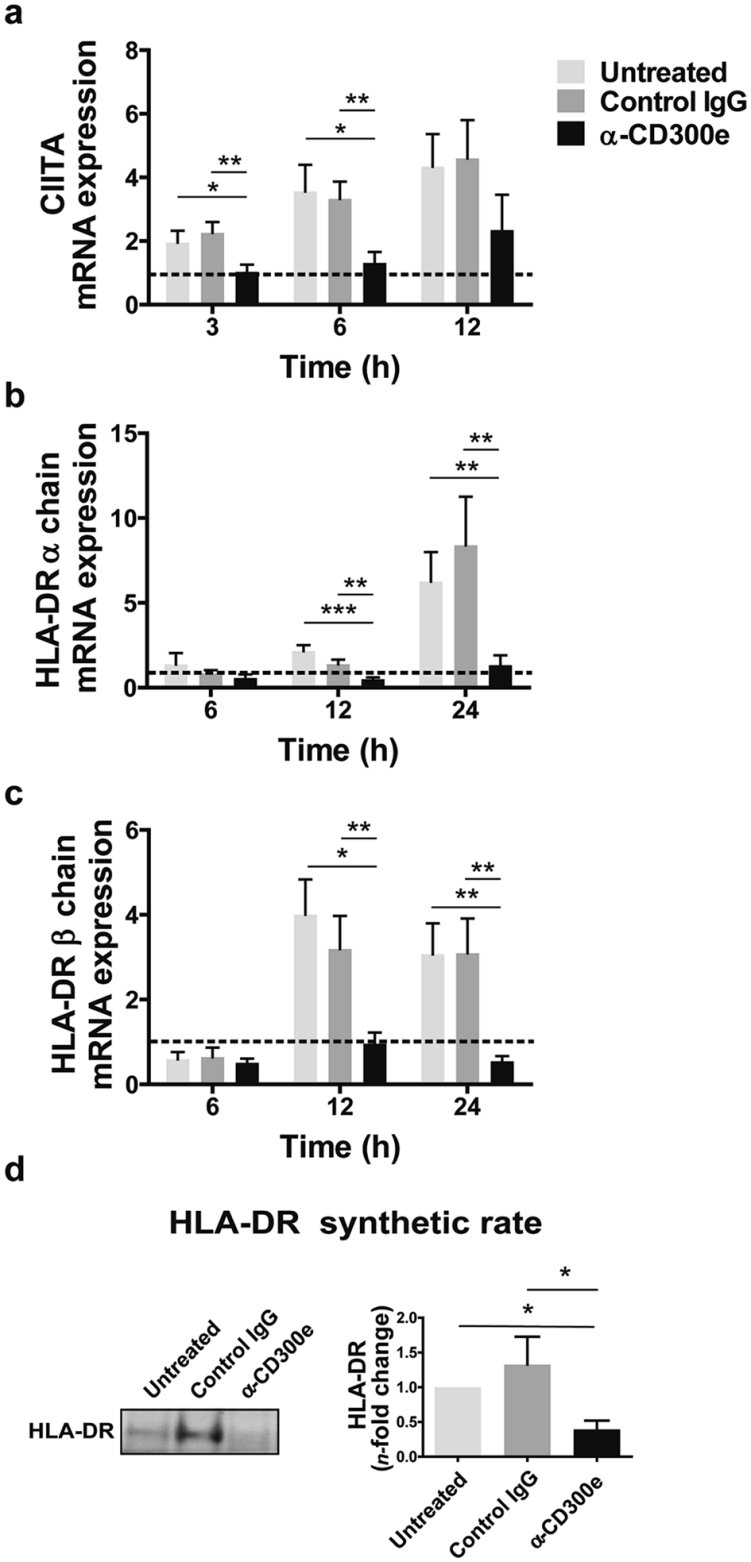
Activation through CD300e affects the synthesis of HLA-II in monocytes. (**a**–**c**) mRNA expression of CIITA, HLA-DR α and β chains in monocytes activated or non-activated through anti-CD300e. Monocytes were plated on 24-well plates uncoated, coated with isotype IgG (control IgG) or with anti-CD300e. At the indicated time points, cells were processed for mRNA extraction. mRNA expression was determined by qRT-PCR and data were normalized to an endogenous reference gene (β-actin). Expression levels of treated cells are relative to values at T_0_ set as 1 (dotted line). Data are shown as mean ± SEM of 4 independent experiments. (**d**) HLA-DR synthetic rate in CD300e-activated or non-activated monocytes. Monocytes were plated on 24-well plates uncoated, coated with isotype IgG (control IgG) or with anti-CD300e. After 24 h, cells were processed as detailed in Methods section. Blot refers to a representative of 3 independent experiments. Quantification of HLA-DR was performed by densitometry and expressed as amount relative to untreated cells set as 1 (mean ± SEM). Significance was determined by Student’s *t*-test. *p ≤ 0.05; **p ≤ 0.01; ***p ≤ 0.001.

Monocytes were treated as above and, after 3, 6 and 12 h, the expression of mRNA for CIITA was determined. Results revealed that, while in control monocytes (either untreated or exposed to the isotypic antibody) the expression of CIITA increased over the time, in cells activated for CD300e it remained at the basal level (Fig. 4a). In accordance with the role of CIITA in the transcription of HLA-II genes, the expression level of mRNAs for HLA-DR, -DP- and -DQ were maintained low in monocytes, following the engagement of CD300e (Fig. 4b,c and Supplementary Fig. S1b,d).

Although this evidence reinforced the notion that the activation through CD300e hampered the expression of HLA-II, the final proof was obtained by comparing the HLA-DR protein synthetic rate in CD300e-activated monocytes vs control monocytes (Fig. 4d).

### CD300e interferes with the IFN-γ pathway

**Figure 5:**
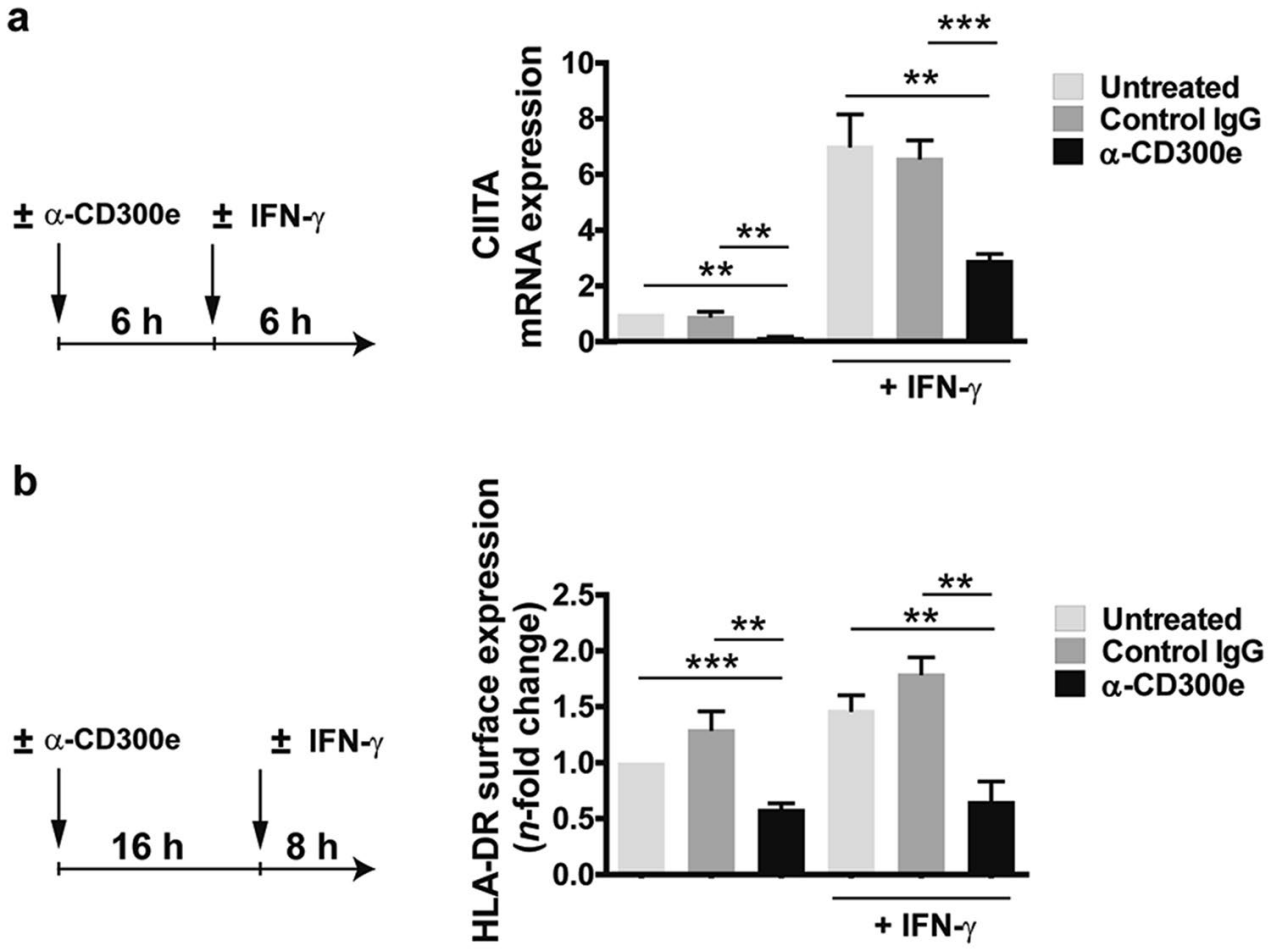
Engagement of CD300e dampens the IFN-γ-induced CIITA and HLA-DR expression. (**a**) mRNA expression of CIITA in monocytes activated or not by anti-CD300e for 6 h and then stimulated for further 6 h with 10 ng/ml IFN-γ. Cells were processed for mRNA extraction. mRNA expression was determined by qRT-PCR and data were normalized to β-actin. Expression levels of treated cells are relative to untreated cells set as 1. Data are shown as mean ± SEM of 4 independent experiments. (**b**) Surface expression of HLA-DR protein in monocytes activated by anti-CD300e for 16 h and then stimulated for further 8 h with 10 ng/ml IFN-γ. Cell surface level of HLA-DR was evaluated by flow cytometry. MFI of HLA-DR ± SEM of 4 independent experiments was calculated and expressed as *n*-fold change relative to untreated cells set as 1. Left panels in (**a**) and (**b**) describe the experimental setup. Significance was determined by Student’s t-test. **p ≤ 0.01; ***p ≤ 0.001.

Interferon-γ (IFN-γ) is a cytokine known to strongly promote the transcription of CIITA and, hence, the expression of HLA-II^16^. Based on the observation that the activation through CD300e maintained the expression of CIITA at low levels in monocytes, we wondered whether this phenomenon was recapitulated even in presence of IFN-γ. Monocytes were stimulated with the anti-CD300e antibody. After 6 h, cells were left on the antibody-coated plate, but the medium was substituted with a fresh one containing IFN-γ and after further 6 h incubation, the expression of mRNA for CIITA was determined. As shown in Fig. 5a, the amount of mRNA for CIITA was up-regulated in control monocytes exposed to the cytokine while it was maintained low in CD300e-activated monocytes, regardless the presence of IFN-γ (Fig. 5a). Accordingly, the ligation of CD300e strongly affected the IFN-γ-induced HLA-DR expression (Fig. 5b). This finding suggests that the signalling pathway triggered by the engagement of the immune receptor could interfere with that activated by IFN-γ.

### Activation through CD300e affects STAT1 expression

**Figure 6:**
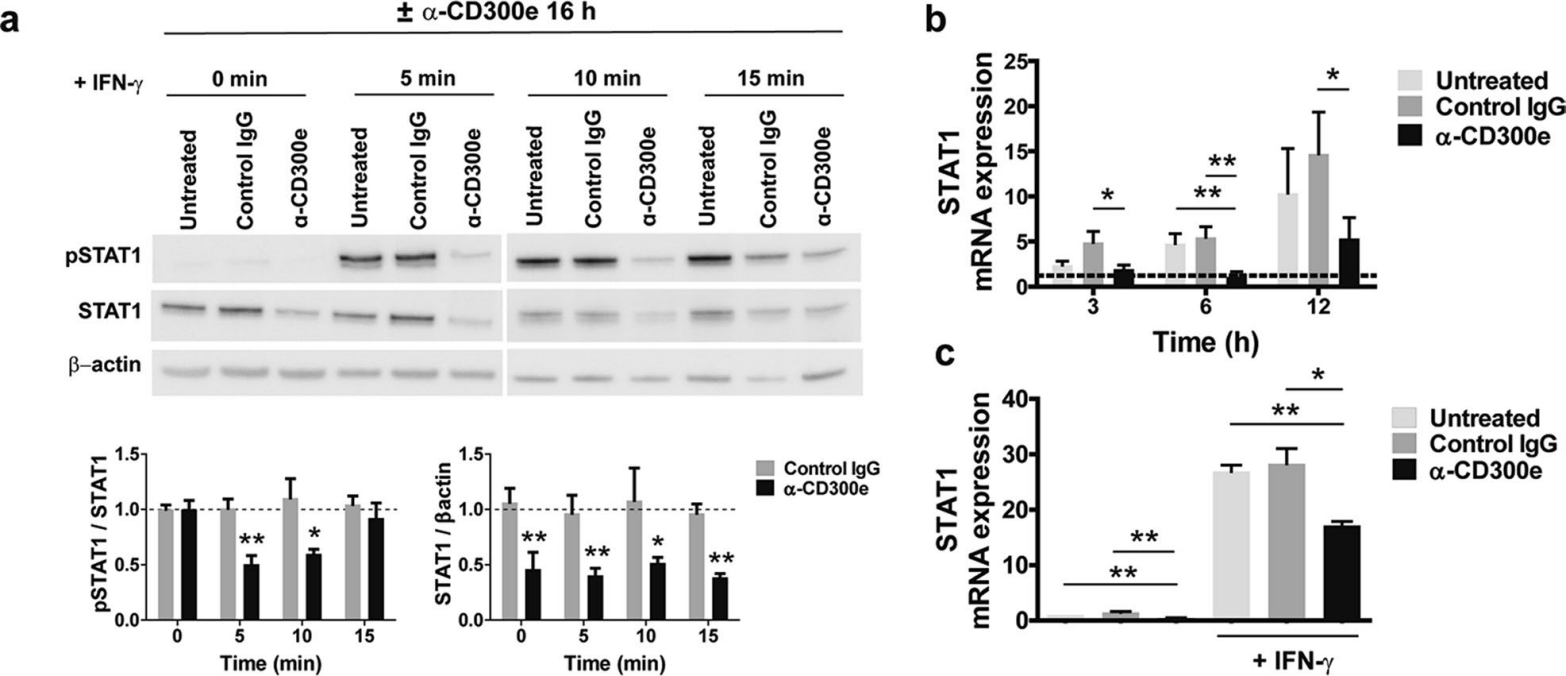
Activation through CD300e impairs STAT1 expression. (**a**) STAT1 and pSTAT1 cell content in monocytes activated or not by anti-CD300e for 16 and then stimulated with 10 ng/ml IFN-γ for 5, 10 or 15 min. Blot refers to a representative of 4 independent experiments. Quantification was performed by densitometry. Data referred to pSTAT1 are expressed as ratio on STAT1; data referred to STAT1 are expressed as ratio of STAT1/β-actin (mean ± SEM). Expression levels of treated cells are relative to values of untreated cells set as 1 (dotted line) for each time point. Significance was determined by Student’s *t*-test vs untreated cells *p ≤ 0.05; **p ≤ 0.01. (**b**) mRNA expression of STAT1 in monocytes activated or non-activated by anti-CD300e. At the indicated time points, cells were processed for mRNA extraction. mRNA expression was determined by qRT-PCR. Data were normalized to β-actin. Expression levels of treated cells are relative to values of T_0_ cells set as 1 (dotted line). Data are shown as mean ± SEM of 4 independent experiment. (**c**) mRNA expression of STAT1 in monocytes activated or not by anti-CD300e for 6 h and then stimulated or not for further 6 h with 10 ng/ml IFN-γ. Cells were processed for mRNA extraction. mRNA expression was determined by qRT-PCR and data were normalized to β-actin. Expression levels of treated cells are relative to untreated cells set as 1. Data are shown as mean ± SEM of 4 independent experiments. Significance was determined by Student’s *t*-test. *p ≤ 0.05; **p ≤ 0.01.

Binding of IFN-γ to its membrane receptor (IFNGR) induces the activation of Janus kinase 1 (JAK1) and 2 (JAK2), which leads to the phosphorylation of the signal transducer and activator of transcription 1 (STAT1) in the cytoplasm. Phosphorylated STAT1 forms a homodimer that translocates into the nucleus and binds to the IFN-γ-activated site (GAS)–E-box motif in the target genes, triggering their expression. Among these genes there is that encoding CIITA^16,17^. To evaluate if the activation through CD300e hindered the phosphorylation of STAT1, monocytes were activated for 16 h on anti-CD300e coated-plate and then stimulated with IFN-γ for 5, 10 and 15 min to induce the activation of STAT1. As shown in Fig. 6a, IFN-γ triggered the phosphorylation of STAT1 in control monocytes, while it was maintained low in cells previously activated through CD300e. However, the most intriguing finding was the reduced amount of unphosphorylated STAT1 in monocytes activated for CD300e, compared to controls. Notably, the IFN- γ -induced phosphorylation of STAT1 was not affected in monocytes that were simultaneously activated through the immune receptor (Supplementary Fig. S4). Collectively, these data support the notion that the CD300e engagement could affect the expression of STAT1 rather than its activation. In concordance with this hypothesis, mRNA for STAT1, which increased over the time in control monocytes, did not augment in CD300e-activated monocytes (Fig. 6b). Even in cells exposed to IFN-γ that stimulates STAT1 gene expression^18^, the level of mRNA was strongly affected by the pre-activation through the immune receptor (Fig. 6c).

### Tissue macrophages express CD300e

In vitro differentiation of human monocytes towards macrophages is accompanied by the downregulation of CD300e^3^. However, in vitro differentiated macrophages represent an oversimplification of tissue resident macrophages which consist of variably mixed populations of embryonic origin and bone marrow-derived blood monocytes. As a result of their complex origin, distribution and responses to endogenous and exogenous stimuli, these cells express marked phenotypic heterogeneity^19,20^.

**Figure 7:**
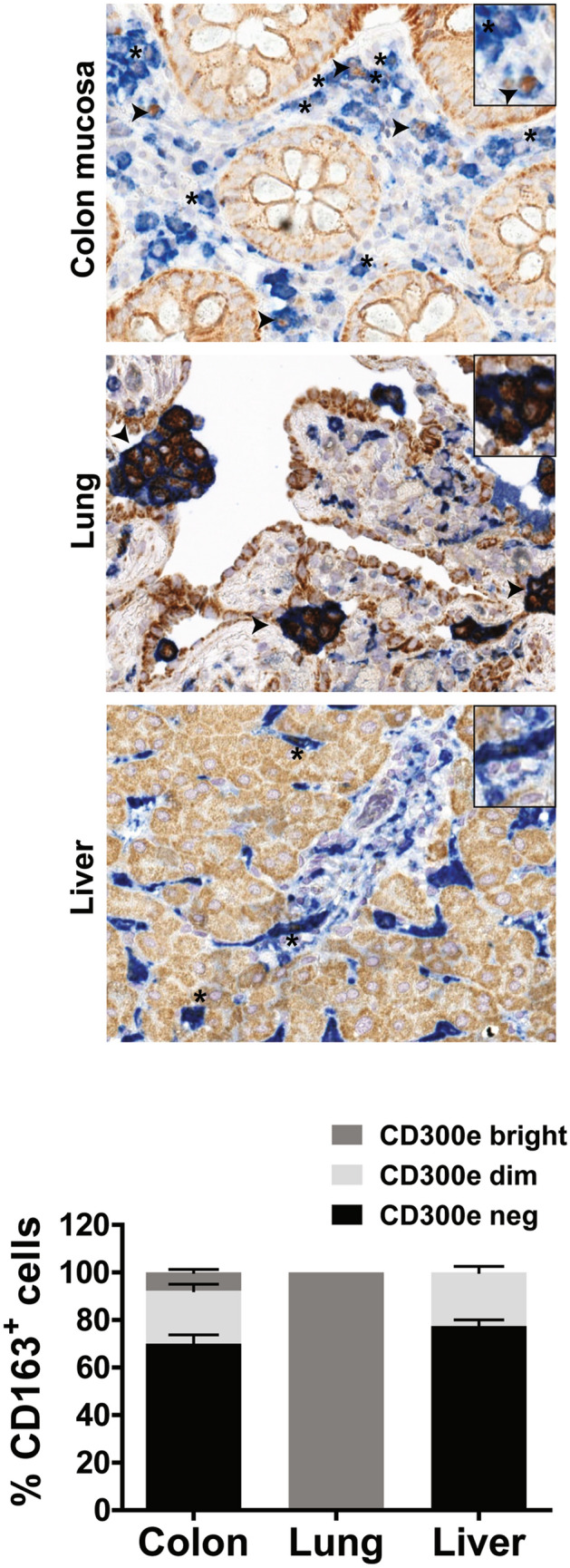
Expression of CD300e in tissue macrophages. Immunohistochemical staining for CD300e (brown) and CD163 (blue) was performed on sections of normal colon mucosa, lung and liver. Arrows highlight macrophages which strongly express CD300e, while asterisks highlight weakly expressing macrophages. The percentage of CD163^+^ macrophages, CD300e^bright^, CD300e^dim^ or not expressing CD300e, in 3 patients for colon mucosa and liver and in 4 patients for lung was calculated and expressed in the bottom plot as mean ± SEM. Original magnification 400× , inset magnification 600 × .

By immunohistochemistry we assessed the expression of CD300e in macrophages infiltrating 3 different tissues, colon mucosa, lung and liver. The analysis revealed that, dependently on the tissue, a different proportion of macrophages, was positive for CD300e (Fig. 7). With respect to the lung, in which all macrophages strongly expressed the immune receptor, in the liver ~ 20% of macrophages were positive for CD300e and displayed a weak pattern of expression. An intermediate scenario was observed in colon mucosa where within the 30–40% of macrophages expressing CD300e it was possible to clumsily distinguish between macrophages with strong expression of CD300e from others with a weak expression. A similar heterogenicity was recapitulated in macrophages infiltrating the adipose tissue from obese subjects (Supplementary Fig. S5). These findings are congruent with those from other studies demonstrating the heterogenous profile of tissue macrophages^19^ and once more confirm that using in vitro differentiated macrophages is a simplistic and inadequate approach to study the functional profile of macrophages.

## Discussion

Most of the immune receptors of the CD300 family, including CD300e, possesses a transmembrane domain and a short cytoplasmic tail. To propagate the downstream signals, the transmembrane domain associates with adaptor proteins containing immunoreceptor tyrosine-based activating motifs (ITAMs)^21^. The two main ITAM-containing adaptors expressed by myeloid cells are DAP12 and FcRγ, the former suggested as adaptor for human CD300e^3^, while both for the mouse homologue^4^. Although for decades ITAM- and ITIM-containing molecules were thought to display opposite functions, this paradigm has been questioned by several evidence showing that the same ITAM-coupled receptor can trigger either an activating or an inhibitory signalling cascade^22–24^.

In line with this finding, we demonstrated in monocytes that CD300e elicits both a positive and a negative signalling pathway. The latter hampers the expression of HLA-II molecules thus, affecting the capacity of the cells to activate a specific T cell response. We are aware that monocytes are minimally involved in the antigen presentation in vivo, with respect to macrophages for example, but they represented an essential cell model to study the pathway triggered by CD300e, since they strongly express the immune receptor. On the other hand, macrophages differentiated from monocytes in vitro lose CD300e^3^.

Interestingly, the ability of CD300e in interfering with HLA-II expression in monocytes overcomes the effect of IFN-γ, potent inducer of antigen-presenting molecules^25^.

**Figure 8:**
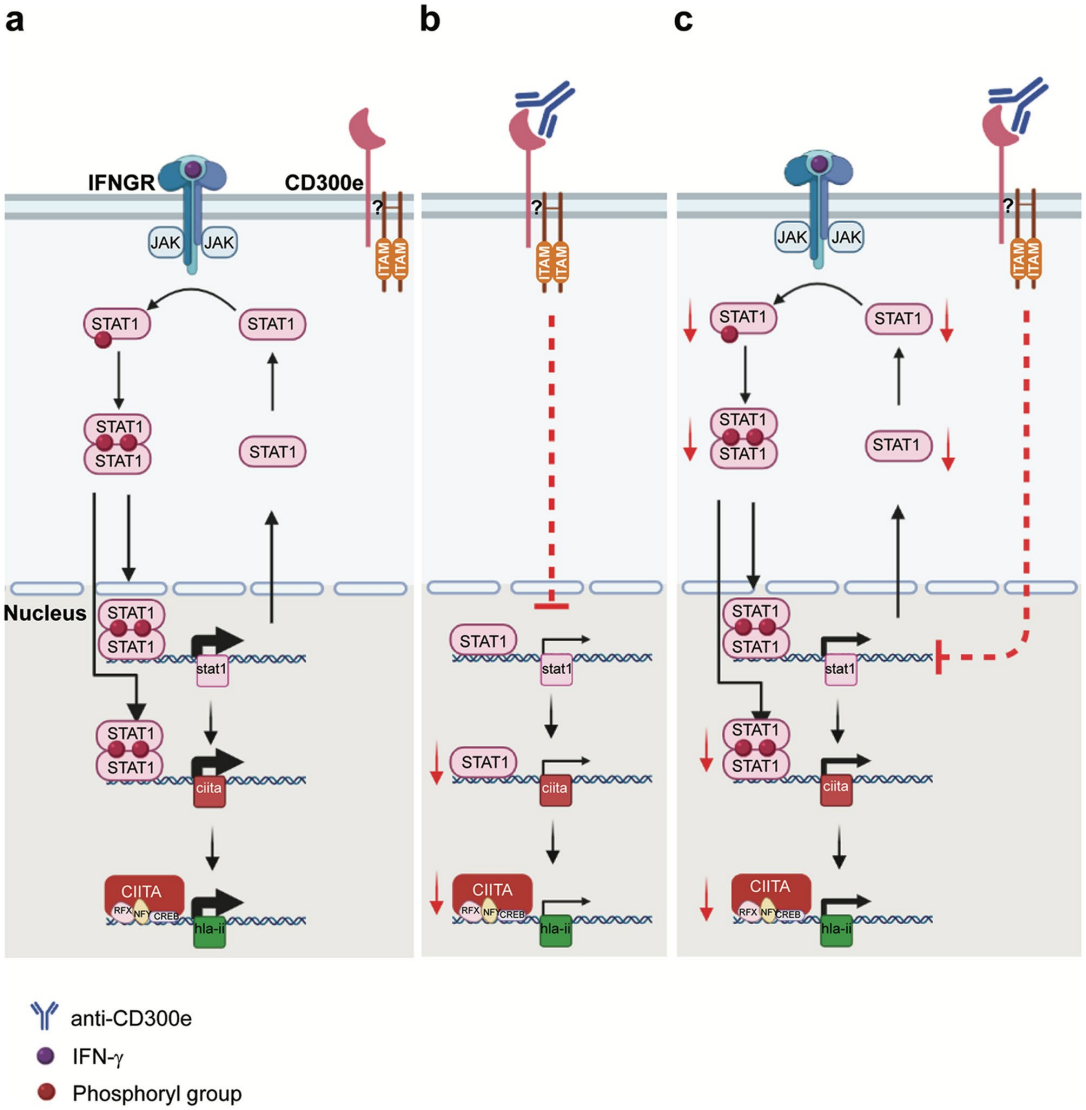
CD300e working model. (**a**) The binding of IFN-γ to the interferon-gamma-receptor (IFNGR) leads to the dimerization of the receptor, activation of JAK1 and JAK2, recruitment and phosphorylation of STAT1. Following dimerization, phosphorylated STAT1 enters the nucleus and transcribes target genes, including STAT1 and CIITA, the master control regulator of HLA-II expression. (**b**) The engagement of CD300e in monocytes thwarts the basal expression of STAT1. Therefore, the unphosphorylated STAT1-dependent expression of CIITA is hampered, and in turn that of HLA-II. (**c**) The activation through CD300e, even under IFN-γ induction, keeps low the transcription of STAT1 gene, thus lowering the amount of STAT1 available for activation by phosphorylation. This, in turn, results in an impairment of CIITA and HLA-II synthesis. RFX, NFY and CREB are part of the heteromultimeric scaffold required for CIITA transcriptional activity.

The IFN-γ-induced HLA-II expression depends on the activation of the STAT1 pathway. Following phosphorylation, STAT1 dimerizes and enters the nucleus. There, it promotes the expression of target genes which include STAT1 itself and CIITA, the master regulator of HLA-II genes (Fig. 8a). Here, we report that the engagement of CD300e in monocytes hinders the expression of STAT1 and CIITA, regardless the presence of the cytokine (Fig. 8b,c). As expected, in cells activated by IFN-γ this leads to a reduction of the amount of STAT1 available for phosphorylation (Fig. 8c).

The existence of an intersection between the signalling cascade triggered by IFN cytokines and that involving receptors associated with ITAM-containing adaptors has been already suggested^26,27^. Several data agree about a positive cooperation between the two signalling cascades which augments the activation of STAT1 and thus the expression of inflammatory target genes^28,29^. Even if the role of the ITAM-containing adaptor DAP12 in the signalling cascade triggered by CD300e remains to be established, our results open the possibility that ITAM-bearing adaptors might also negatively modulate the STAT1 pathway activated by IFN-γ.

It is worthy to highlight that CD300e affects the expression of STAT1, and that of its target CIITA, even in the absence of IFN-γ. Although this may sound curious because the expression of STAT1 target genes is known to require the transcription factor in its phosphorylated form, it has been demonstrated that unphosphorylated STAT1 mediates the basal expression of several target genes^30^, including CIITA and STAT1^31^ (Fig. 8b).

Despite in vitro-differentiated macrophages do not express CD300e, we got preliminary evidence that the immune receptor is actually expressed by a proportion of tissue macrophages. Moreover, in accordance with the notion that tissue macrophages are a heterogenous population, with different function, we want to emphasize the evidence that CD300e is not equally expressed by all tissue macrophages and varies among different tissues. The fact that CD300e could modulate the antigen presentation capacity also in tissue macrophages and in other antigen presenting cells, such as dendritic cells, is an aspect that still needs to be further investigated. Furthermore, the evidence of a strong expression of CD300e by the glands of colon mucosa and by pneumocytes opens the possibility that the immune receptor could have a function also in non-immune cells.

Conscious that, beside the identification of the ligand of CD300e, many mechanistic details are still missing, including if truly CD300e cooperates with an ITAM-bearing protein adaptor and how its activation inhibits the synthesis of STAT1, we propose the existence of a pathway triggered by CD300e that negatively modulates the expression of STAT1.

Overall, our data support the notion that CD300e is not an activating receptor sensu stricto and open the possibility that this receptor might be a novel immune check point that contributes to the regulation of the expansion of T cell-mediated responses. Moreover, the induction of the immune receptor expression on the surface of phagocytic cells could represent a strategy for bacterial pathogens to evade the host immune response, as already suggested for *Helicobacter pylori*^7^ .

## Supplementary information

Supplementary Figures.

## Data Availability

The data that support the plots and other findings of this study are available from the corresponding author upon reasonable request.

## References

[1] Borrego F (2013). The CD300 molecules: an emerging family of regulators of the immune system. Blood.

[2] Brckalo T (2010). Functional analysis of the CD300e receptor in human monocytes and myeloid dendritic cells. Eur. J. Immunol..

[3] Aguilar H (2004). Molecular characterization of a novel immune receptor restricted to the monocytic lineage. J. Immunol..

[4] Isobe M (2018). The CD300e molecule in mice is an immune-activating receptor. J. Biol. Chem..

[5] Zenarruzabeitia O (2016). The expression and function of human CD300 receptors on blood circulating mononuclear cells are distinct in neonates and adults OPEN. Nat. Publish. Group.

[6] Hey Y-Y, O’Neill TJ, O’Neill HC (2019). A novel myeloid cell in murine spleen defined through gene profiling. J. Cell Mol. Med..

[7] Pagliari M (2017). Helicobacter pylori affects the antigen presentation activity of macrophages modulating the expression of the immune receptor CD300E through miR-4270. Front. Immunol..

[8] D’Elios MM (2017). The Helicobacter cinaedi antigen CAIP participates in atherosclerotic inflammation by promoting the differentiation of macrophages in foam cells. Sci. Rep..

[9] Lu HK (2009). Leukocyte Ig-like receptor B4 (LILRB4) is a potent inhibitor of FcγRI-mediated monocyte activation via dephosphorylation of multiple kinases. J. Biol. Chem..

[10] Walseng E, Bakke O, Roche PA (2008). Major histocompatibility complex class II-peptide complexes internalize using a clathrin- and dynamin-independent endocytosis pathway. J. Biol. Chem..

[11] D’Elios MM (1997). Predominant Th1 cell infiltration in acute rejection episodes of human kidney grafts. Kidney Int..

[12] Liu E, Tu W, Law HKW, Lau YL (2001). Changes of CD14 and CD1a expression in response to IL-4 and granulocyte-macrophage colony-stimulating factor are different in cord blood and adult blood monocytes. Pediatr. Res..

[13] Xiao, J. & Chen, H. S. (2005) Biological functions of melanoma-associated antigens (MAGEs) in cell activities. *Chin. J. Cancer* **24**, 124–128 15642216

[14] Pinet V, Vergelli M, Martini R, Bakke O, Long EO (1995). Antigen presentation mediated by recycling of surface HLA-DR molecules. Nature.

[15] Furuta K, Walseng E, Roche PA (2013). Internalizing MHC class II-peptide complexes are ubiquitinated in early endosomes and targeted for lysosomal degradation. Proc. Natl. Acad. Sci. USA.

[16] Reith W, LeibundGut-Landmann S, Waldburger J-M (2005). Regulation of MHC class II gene expression by the class II transactivator. Nat. Rev. Immunol..

[17] Muhlethaler-Mottet A, Berardino WD, Otten LA, Mach B (1998). Activation of the MHC class II transactivator CIITA by interferon-γ requires cooperative interaction between Stat1 and USF-1. Immunity.

[18] Lehtonen, A., Matikainen, S. & Julkunen, I. Interferons up-regulate STAT1, STAT2, and IRF family transcription factor gene expression in human peripheral blood mononuclear cells and macrophages. *J. Immunol. (Baltimore, Md. : 1950)* **159**, 794–803 (1997). 9218597

[19] Gordon S, Plüddemann A (2017). Tissue macrophages: heterogeneity and functions. BMC Biol..

[20] Italiani, P., Boraschi, D. & Ley, K. From monocytes to M1/M2 macrophages: phenotypical vs. functional differentiation. (2014) 10.3389/fimmu.2014.00514. 10.3389/fimmu.2014.00514 PMC4201108 25368618

[21] Humphrey MB, Lanier LL, Nakamura MC (2005). Role of ITAM-containing adapter proteins and their receptors in the immune system and bone. Immunol. Rev..

[22] Turnbull IR, Colonna M (2007). Activating and inhibitory functions of DAP12. Nat. Rev. Immunol..

[23] Hirsch I, Janovec V, Stranska R, Bendriss-Vermare N (2017). Cross talk between inhibitory immunoreceptor tyrosine-based activation motif-signaling and toll-like receptor pathways in macrophages and dendritic cells. Front. Immunol..

[24] Blank U, Launay P, Benhamou M, Monteiro RC (2009). Inhibitory ITAMs as novel regulators of immunity. Immunol. Rev..

[25] Boehm U, Klamp T, Groot M, Howard JC (1997). Cellular responses to interferon-γ. Annu. Rev. Immunol..

[26] Bezbradica JS, Rosenstein RK, Demarco RA, Brodsky I, Medzhitov R (2014). A role for the ITAM signaling module in specifying cytokine-receptor functions. Nat. Immunol..

[27] Ivashkiv LB (2009). Cross-regulation of signaling by ITAM-associated receptors. Nat. Immunol..

[28] Wang L (2008). ‘Tuning’ of type I interferon-induced Jak-STAT1 signaling by calcium-dependent kinases in macrophages. Nat. Immunol..

[29] Tassiulas I (2004). Amplification of IFN-α-induced STAT1 activation and inflammatory function by Syk and ITAM-containing adaptors. Nat. Immunol..

[30] Michalska, A., Blaszczyk, K., Wesoly, J. & Bluyssen, H. A. R. A positive feedback amplifier circuit that regulates interferon (IFN)-stimulated gene expression and controls type I and type II IFN responses. *Front. Immunol. 9*, (2018). 10.3389/fimmu.2018.01135 PMC5985295 29892288

[31] Chatterjee-Kishore M, Wright KL, Ting JPY, Stark GR (2000). How Stat1 mediates constitutive gene expression: a complex of unphosphorylated Stat1 and IRF1 supports transcription of the LMP2 gene. EMBO J..

