# Supplementary material for: The immune receptor CD300e negatively regulates T cell activation by impairing the STAT1-dependent antigen presentation
Source: Sci Rep. 2020 Oct 5;10:16501. doi: 10.1038/s41598-020-73552-9 (PMC7536427; doi:10.1038/s41598-020-73552-9)
Supplement: Supplementary file 1 — Supplementary Figures. [file 41598_2020_73552_MOESM1_ESM.docx]

# **The immune receptor CD300e negatively regulates T cell activation by impairing the STAT1-dependent antigen presentation**

Sara Coletta, Valentina Salvi, Chiara Della Bella, Ambra Bertocco, Silvia Lonardi, Elisabetta Trevellin, Matteo Fassan, Mario M. D’Elios, William Vermi, Roberto Vettor, Stefano Cagnin, Silvano Sozzani, Gaia Codolo and Marina de Bernard

# **Supplementary Figures**

**Supplementary Figure S1. Activation through CD300e reduces the expression of HLA-DP and -DQ in monocytes.** (a and c) Flow cytometry analysis of the cell surface HLA-DP and -DQ. Monocytes were plated on 24-well plates uncoated, coated with isotypic IgG (control IgG) or with anti-CD300e monoclonal IgG (UP-H2). After 24 h, median fluorescence intensity (MFI) of HLA-DP or -DQ ± SEM of 4 independent experiments was calculated. Expression levels of treated cells are expressed as *n*-fold change relative to untreated cells set as 1 (dotted line). Significance was determined by Student’s *t*-test vs untreated cells. **p≤ 0.01. (b and d) mRNA expression of HLA-DP or HLA-DQ (α and β chains) in monocytes activated or non-activated by anti-CD300e. Monocytes seeded as above were processed for mRNA extraction. mRNA expression was determined by qRT-PCR and data were normalized to an endogenous reference gene (β-actin). Expression levels of treated cells are relative to values at T_0_ set as 1 (dotted line). Data are shown as mean ± SEM of 4 independent experiments. Significance was determined by Student’s *t*-test. *p≤ 0.05; **p≤ 0.01.

**Supplementary Figure S2. Activation through CD300e leads monocytes to acquire a pro-inflammatory profile with a macrophage-like phenotype.** Monocytes were seeded on uncoated, control IgG- (isotype control) or anti-CD300e-coated plates for 24 h. (a-d) Flow cytometry analysis of HLA-DR, CD86, CD68 and CD206 expression. Expression levels of treated cells are expressed as *n*-fold change relative to untreated cells set as 1 (dotted line). Data are shown as mean ± SEM, n=4. Significance was determined by Student’s *t*-test vs untreated cells. *p≤ 0.05; **p≤ 0.01; ***p≤ 0.001. (e-h) IL-1β, IL-6, IL-8 and TNF-α protein content in the culture supernatants was determined by ELISA assay. Data are expressed as mean ± SEM, n=5. Significance was determined by Student’s *t*-test. *p≤ 0.05; **p≤ 0.01.

**Supplementary Figure S3. The capacity to down-regulate the expression of HLA-DR does not involve released factors and is a distinctive feature of CD300e.** (a) Freshly isolated monocytes were exposed to a conditioned medium of monocytes seeded on uncoated, isotype IgG or anti-CD300e-coated plates for 24 h. Cell surface level of HLA-DR (upper panels) and HLA-DR total cell content (lower panels) were evaluated after 24 h by flow cytometry and western blot, respectively. (b) Conditioned medium of monocytes seeded on uncoated, isotype IgG or anti-CD300e-coated plates for 24 h was depleted of IgG with protein G agarose before being transferred on freshly isolated monocytes. Antibody depletion was performed to ruled out the possibility that the observed effect was due to the presence of anti-CD300e antibody harvested during medium collection. After 24 h, cell surface level of HLA-DR (upper panels) and HLA-DR total cell content (lower panels) were evaluated by flow cytometry and western blot, respectively. Right upper panels in (a) and in (b) are representative FACS histograms. Median fluorescence intensity (MFI) of HLA-DR ± SEM of 4 independent experiments was calculated and expressed as *n*-fold change relative to cells incubated with medium of cells plated on uncoated plastic set as 1 (dotted line). Significance was determined by Student’s t-test vs untreated cells. *p≤ 0.05. Blots in (a) and (b) are representative of 4 independent experiments. Quantification of HLA-DR was performed by densitometry and normalized to vinculin (mean ± SEM). Significance was determined by Student’s t-test vs control IgG. *p≤ 0.05. (c) CD64 molecules were cross-linked on freshly isolated monocytes. Cells were seeded on plates coated with F(ab′)_2_ fragment only or on plates coated with F(ab′)_2_ fragment plus isotype IgG or anti-CD64 antibodies. The level of HLA-DR surface expression (left panel) and the amount of TNF-α in the culture supernatants (used as positive control for CD64 activation- right panel) were determined after 24 h by flow cytometry and ELISA assay, respectively. Median fluorescence intensity (MFI) of HLA-DR was expressed as *n*-fold change relative to cells seeded on F(ab′)_2_ fragment-coated plates set as 1 (dotted line). Data are shown as mean ± SEM of 3 independent experiments. Significance was determined by Student’s t-test. ***p≤ 0.001.

**Supplementary Figure S4. Activation through CD300e does not interfere with the phosphorylation in case of simultaneous stimulation with IFN-γ.** The levels of pSTAT1 and STAT1 were evaluated by western blot in monocytes exposed simultaneously to PBS (untreated), isotype IgG (control IgG) or anti-CD300e and 10 ng/ml IFN-γ for 5, 10 or 15 min. Beta-actin was used as loading control.

**Supplementary Figure S5. Expression of CD300e in macrophages infiltrating adipose tissue.** Stromal vascular fraction isolated from subcutaneous adipose tissue of 3 obese patients were labelled with anti-CD68-PE and anti-CD300e antibody, followed by anti-mouse Alexa Fluor 488 and analysed by flow cytometry (upper panel). Expression of CD300e was evaluated in the CD68^+^ cell population. The percentage of CD68^+^ macrophages, CD300e^bright^, CD300e^dim^ or not expressing CD300e, was calculated and expressed in the bottom panel.

# **Full-length blots**

**Fig. 3b**

**Fig. 4d**

**Fig. 6a**

**Supplementary Figure S3a and b**

**Supplementary Figure S4**
